# Orofacial Granulomatosis in a Child

**DOI:** 10.1155/2019/7519267

**Published:** 2019-12-03

**Authors:** Reena Razdan, Maxwell D. Newby, Michele M. Carr

**Affiliations:** ^1^West Virginia University School of Medicine, Morgantown, WV, USA; ^2^West Virginia University Department of Otolaryngology, Morgantown, WV, USA

## Abstract

Orofacial granulomatosis (OFG) is a rare, idiopathic disorder of the orofacial region. It is clinically characterized by persistent and/or recurrent enlargement of the soft tissues of the oral and maxillofacial region, often manifesting as labial enlargement and swelling of intraoral sites such as the gingiva, tongue, and buccal mucosa. Full-thickness mucosal biopsy reveals noncaseating granulomatous inflammation, similar to Crohn's disease and sarcoidosis. Thus, OFG must be distinguished from other chronic granulomatous disorders. We report a case of a young female patient who presented with labial and maxillary gingival enlargement without any identifiable systemic causes, with suggested involvement of environmental triggers.

## 1. Introduction

The term orofacial granulomatosis (OFG) was first coined by Wiesenfeld et al. in 1985 to describe a rare granulomatous inflammation in the orofacial area in the absence of a systemic condition [1, 2]. Clinically, OFG presents as lip swelling often with oral involvement, including the gingiva, tongue, and buccal mucosa, without a clear origin of pathophysiology [2]. Histologically, the condition is marked by noncaseating granulomas with lymphocytic infiltrate and epithelioid histiocytes [3]. Perivascular aggregation of histiocytes and plasma cells may be observed along with dilated lymphatics in the superficial lamina propria [3]. Early medical intervention is essential in preventing and minimizing permanent swelling, functional impairment in speaking and eating, and emotional distress from aesthetic appearance [2].

## 2. Case Presentation

A 9-year-old Caucasian female presented to the West Virginia University otolaryngology clinic with the chief complaint of gradually increasing swelling of her upper and lower lips which started 4 months prior. The lip swelling was reported to be very distressing to the child and her parents. Prior to presentation at the otolaryngology clinic, the patient was seen by a pediatric dentist, periodontist, and allergist. The patient had a history of excessive thumb sucking, wearing nail polish, and playing with homemade slime containing glue, saline, shaving cream, and glitter. The patient had a history of mild eczematous skin lesions and vague rhinitis including nasal congestion, occasional postnasal drainage, varying complaints of headaches, and occasional conjunctival pruritus. Seasonal symptoms were worse in spring time, with the pollens of trees, grasses, and weeds possibly playing a role. There was no contributory family history, medication history, or history of allergy to food, drugs, or cosmetics. The patient did not have any contributory past medical or surgical history.

**Figure 1:**
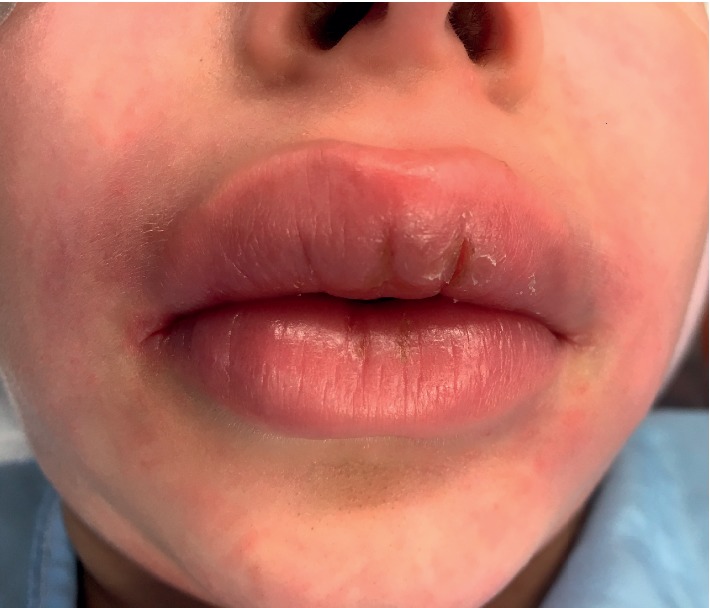
Prominent upper lip swelling with fissures that had persisted for four months.

**Figure 2:**
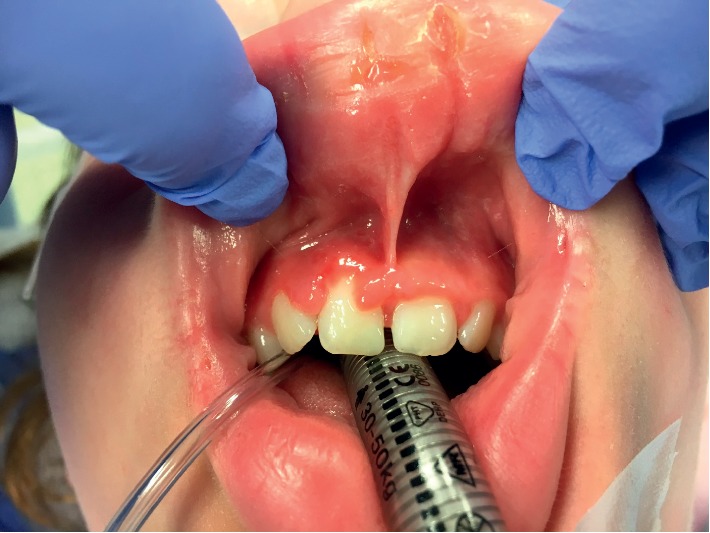
Maxillary gingival hypertrophy associated with lip swelling.

Physical examination revealed a symmetrically enlarged, erythematous upper lip with erythematous and edematous maxillary anterior gingiva, particularly on the right side (Figures 1 and 2). The allergist also noted mild fissures in the lip area, as well as evidence of cheilitis. The patient was prescribed mupirocin ointment for the perioral areas where the cheilitis was present in order to clear any superficial infection. This treatment likely resolved these issues prior to patient presentation at the otolaryngology clinic.

The suggested differential diagnosis was Crohn's disease, sarcoidosis, tuberculosis, angioedema, and orofacial granulomatosis. Routine laboratory tests (basic metabolic panel, complete blood count with differential, erythrocyte sedimentation rate, hepatic function panel, HAV antibody, HCV antibody, HBV envelope antibody and antigen, C-reactive protein, immunoglobulin panel, C4 complement, C3 complement, glomerular basement membrane antibodies, and cytoplasmic neutrophil antibodies) were performed and were reported to be within normal limits. No clinical signs or symptoms of gastrointestinal disease were present.

A right upper lip incisional biopsy was performed. Submucosal lymphohistiocytic infiltrate with loose perivascular granulomas were found. The underlying salivary gland tissue was prominent with lymphocytic infiltrate with occasional germinal center formation. Acid-fast bacilli were not seen on a concentrated smear. The patient's history, physical examination, blood and serum tests, and pathology results led to a diagnosis of OFG of the upper and lower lip with maxillary gingival hyperplasia.

The patient was treated with azithromycin pulses for one month. This treatment consisted of 500 milligrams once daily for 3 days, followed by 4 days of no medication. This regimen was followed for a total of 4 weeks as previously described by De et al. in an effort to avoid corticosteroids given the parents' concern for corticosteroid side effects [1]. The patient saw improvement of symptoms following this treatment. However, after using a lip gloss containing glitter, she experienced an exacerbation of her oral swelling and returned to the clinic. Given the perceived treatment failure, the patient was subsequently treated with 40 milligrams once daily of prednisone for 2 weeks. The patient reported marked improvement following oral steroid administration. Once again, however, the patient experienced an exacerbation of her OFG following swimming. She was treated again with the same regimen of oral steroids. In addition, she avoided foods containing benzoates as well as products containing glitter, both of which seemed to be triggers for her OFG. The patient experienced noticeable improvement in her gingival hyperplasia and lip swelling following the treatment with oral steroids and behavioral modifications.

## 3. Discussion

OFG is a diagnosis of exclusion. A negative history of gastrointestinal or pulmonary complaints precludes the need for further consideration of Crohn's disease or sarcoidosis, respectively [3]. The patient did present with many symptoms of allergic angioedema including nonpitting edema of the lips and face and history of atopic disease (e.g., vague rhinitis and mild eczema) [3]. However, granulomatous inflammation is not a feature of angioedema, and thus this condition could be excluded [2]. Stains of the biopsy specimen for acid-fast bacilli ruled out mycobacterial infection. Thus, a diagnosis of OFG could be made with negative findings for other possible conditions. The patient was referred to pediatric rheumatology for continued treatment.

The etiology and pathogenesis of OFG is unknown. Many studies suggest a delayed hypersensitivity reaction with an unlikely genetic component [3]. Several studies have suggested a link between OFG and food allergy, allergy to dental materials, and inappropriate immunological response [3, 4]. In this patient's case, there appeared to be clear triggers for exacerbation of labial swelling. Her excessive thumb sucking may not only exposed the oral region to irritants, but may also caused trauma in the area that contributed to excess swelling. The patient also chose to eliminate benzoates, often found in food preservatives, from her diet. Diets free of cinnamon and benzoates have been shown to provide benefit in 54–78% of patients diagnosed with OFG, with 23% requiring no adjunctive therapy [5]. However, Campbell et al. more broadly suggested that OFG is a heterogeneous condition where various triggers exist that induce a similar inflammatory response in the orofacial region, and dietary intervention as a monotherapy is not recommended [6]. It is unclear if the benzoate- or glitter-containing products were the primary cause of OFG or simply exacerbated the existing disease process. However, the slow onset of symptoms does support a possible etiology of delayed hypersensitivity reaction. Regardless, identification of contributing factors and minimizing exposure to these triggers have been proven to maximize treatment efficacy. This presents both the limitation and strength of this case report. The temporal connections of the facial swelling and possible environmental triggers increases the evidence of immunological pathogenesis. This finding could allow more conservative treatment modalities in environmental control and decrease the use of antibiotics, corticosteroid, and other immunological modulators. The limitation of this study is the patient was not tested for immunological responses to the specific suspected triggers nor were the triggers eliminated sequentially. Therefore, it is unknown if avoidance of environmental triggers or corticosteroids was the effective treatment. Another alternative would be that the two therapies worked synergistically achieving the favorable response. Rapid resolution of symptoms was the goal of her management. While this practice disallowed identification of a sole trigger, we felt the clinical improvement of the patient provided strong enough evidence to support the avoidance of these irritants.

Early diagnosis and management of OFG is essential in preventing permanent manifestations of the condition. Repeated bouts of labial edema can result in permanent induration and swelling that can functionally impair a patient's ability to speak or eat, as well as cause a great deal of emotional distress from facial disfigurement [7, 8]. Spontaneous remission of OFG is unlikely [3]. It is important to continue monitoring patients and to treat with corticosteroids in order to prevent permanent change. Steroids have been shown to be effective in reducing and preventing recurrence of swelling [3].

Patients diagnosed with OFG are at risk to develop other symptoms and conditions later. In particular, OFG has been shown to be an early manifestation of Crohn's disease, which may present with gastrointestinal symptoms years later [9]. OFG can also be the first presenting symptom of Melkersson–Rosenthal Syndrome (MRS) [10]. This disease manifests as a triad of recurrent labial enlargement, fissured tongue, and lower motor neuron facial palsy; histopathology is consistent with OFG [3]. OFG is often considered a variant and monosymptomatic form of MRS. Consequently, the association between OFG and other conditions warrants persistent clinical surveillance for the development of additional signs and symptoms.

## 4. Conclusion

OFG should be considered in the differential diagnosis for nonspecific labial swelling in the absence of systemic disease. Thorough history, physical exam, and laboratory investigation should be performed to exclude other etiologies. Immediate treatment of OFG can prevent permanent deformity in the orofacial region. Corticosteroids are considered the mainstay treatment for OFG. However, individual patient reports of dietary or environmental triggers should be taken into consideration. The presented case highlights that avoidance of reported triggers can help minimize corticosteroid therapy. In addition, continued monitoring is advised since OFG may be a precursor to other related conditions.
